# To Junior Dentists

**Published:** 1886-02

**Authors:** 


### Editorial.

### TO JUNIOR DENTISTS. NO. IV.

### THE TREATMENT OF DEVITALIZED TEETH.

*My Dear Doctor:*

If there be one dentist who makes any pretentions to being an operator and who has not a special system of treatment for dead teeth, and his own exclusive method of manipulation for the particular material with which to fill roots, I have not yet made his acquaintance. I confess that I have mine, but you cannot imagine the reluctance with which I attempt to give it in detail. The subject, so old and yet so new, has become hackneyed, and so much of what our English brethren call "rot" has been said and written about it, that it seems impossible for me to give you anything that shall prove either interesting or instructive; and yet, upon the proper and successful treatment of these teeth will, to a very large extent, depend your professional future. Almost any man can save a good tooth, but it takes one possessed of knowledge and skill to save one that is badly diseased. Physicians make or lose their reputation by their treatment of critical cases, and not by the cure of simple colds or inflammations. So a dentist will gain fame, not by the filling of simple cavities or the manufacture of rubber plates, but by restoring to usefulness organs that others have condemned to the forceps. Our calling has arrived to such a pitch of perfection that, extraordinary complications alone excepted, the salvation of any tooth or root is simply a question of relative skill and knowledge.

If your neighbor is competent to successfully treat all classes of diseased teeth save one, and you have the ability to save even those, depend upon it the people will find it out and you will gain a clear lead, even though such a case may not present itself once a quarter. Of course you desire to be the first in your field, and it therefore behooves you to carefully study the difficult cases. You wish to succeed where others declare that success is impossible, but this cannot be hoped for without a clear comprehension of all the conditions, and an intimate knowledge of your materia medica. Specific rules and recipes and formulas will not help you. That is the quack method of practice. You must be competent to make your own formulas, and to devise your own methods, founded upon a perfect diagnosis of the case. Physicians cannot aid you in the treatment of dental tissue. They are usually the worst of advisers, for their training would lead them to extirpate that which they cannot comprehend, and a medical education teaches nothing concerning the treatment of teeth. Medical men will insist upon the immediate removal of a diseased tooth as a thing that it is hopeless to expect to return to a state of usefulness. Witness the ineffable bosh that has appeared in some medical journals about dead teeth being foreign substance in the jaws. I shall, therefore, write to you chiefly about principles and laws, for when these are comprehended the question of manipulation and materials will readily adjust itself.

Of course you understand the minute structure of the teeth and their surroundings. If you do not you have no right to call yourself a dentist, and should return to your studies at once. In the lectures to which you listened at college, these matters were all made plain to you But are you sure that you have a *practical* acquaintauce with them? Theory is all very well, but do you comprehend what the pathological condition is, and the principles involved in bringing about a return to a true physiological state? Of course I cannot, within the compass of such a letter as this, attempt a full exhibition of the whole theory of practice. I desire rather to set you to thinking, and to induce you to make a practical application of that which you now know.

What is a so-called dead tooth? Is it in truth a foreign substance, as the name would denote, or have we a misnomer in our lexicon? If it be in the same condition with dead bone, then extraction is the only cure. But it is not, for every competent dentist is constantly saving teeth that are called "dead." Many and many of our patients are wearing and subjecting to constant use "dead" teeth. When an organ or tissue is in the true sense of the term dead, the sooner it is removed the better. But teeth that are called dead do not in any way call for extraction.

We commonly speak of a tooth as dead when the pulp is devitalized. But the pulp does not comprise all the life there is in a tooth. You know that the hard portion is composed of three tissues; enamel, dentine, and cementum. The enamel contains comparatively little of vitality or circulation. There is scarce any change in it after its eruption, and its nutrition is, relatively speaking, nothing. It is not in contact with any soft tissue, and therefore it may be left out of the consideration of living dental structure. The only portions of the tooth that can exercise a malign influence when the pulp is destroyed are the dentine and the cementum. Of these the dentine only is wholly dependent upon the pulp for nutrition, for the cementum is nourished by the periosteum. The dentine alone can in any sense be properly considered as dead. When the pulp is devitalized, the cementum has still its source of nutriment, while the enamel does not particularly need nourishment. If, then, the investing membrane of a tooth be in a healthy state, the dentine is the only tissue that needs fortification and protection when the pulp dies, and this only from attacks that proceed from the pulp-chamber or nerve canals, for its exterior surface is fully protected by the cementum and enamel which closely invest it in every direction. The dentinal fibrillæ are cut off from their living connections, and must accommodate themselves to new conditions. How shall this be brought about?

Did you ever read Tyndall's "Floating Matter of the Air," or Pasteur's "Researches upon Putrefaction," or Magnin's "Bacteria"? If so, you will have some comprehension of what it is necessary to accomplish in preventing that condition which is called "sepsis." If you have not, let me entreat you at least to make a study of the first of these. I shall not go into a consideration of the bacterian theory. It is sufficient to know that if we can secure and maintain a condition of antisepsis, there will be no decay or putrefaction of animal matter. This may be accomplished in two ways: First, by the use of antiseptics; secondly, and more effectually, by the exclusion of all atmospheric conditions, or communication with the air, which is constantly loaded with the germs of fermentive and putrefactive organism. Of course you have a knowledge of the character and qualities of those remedies which are called "antiseptics," and of that peculiar condition called "septic." I am obliged to presuppose this knowledge, because, even if you are not thoroughly informed of the latest advances of science in this direction, this is no place, nor have I the time or space to enter upon their consideration.

And now for the practical application of some of these principles. When the pulp of a tooth is devitalized (not when the *tooth* is dead).

that tissue is cut off from all nutritive sources and is ready for putrefactive changes as soon as the septic organism may be introduced. It may be that if there is no break in the continuity of the enamel or dentine the pulp will not putrefy, but will become desiccated or dried, and will remain in a peaceful state for an indefinite length of time. But usually putrefaction at once sets in, and the resulting gases being forced through the foraminal opening establish an inflamed and septic condition, which results in alveolar abscess. Remember that this arises from putrefaction of the pulp, or from that of matter which may have become infiltrated into an open pulp-chamber or nerve canal. How is this to be prevented?

If, upon the death of a pulp and before any putrefactive changes shall have taken place, the pulp chamber be carefully opened and all traces of animal matter be removed, of course there would be nothing to putrefy and a septic condition could not be set up. And if the pulp chamber and the nerve-canal or canals could now be hermetically sealed against the intrusion of any foreign matter, this condition could be maintained indefinitely. But, practically, both these results are impossible. We cannot extract all the dead tissue, nor can we hermetically seal under such circumstances. We are obliged to resort to antiseptics to destroy all germs and to put any remaining particles of putrefactive material in such a condition that sepsis cannot occur. We must first get out all the remains of the pulp as far as is possible. To do this, it is absolutely essential that we should have the most direct access to the cavity possible. You cannot remove a pulp through a hole at the side of a tooth. You cannot get it out through a cavity upon the posterior surface of any of the back teeth. In the latter case you must open up the cavity until a broach can be thrust directly down into the nerve canal. You will absolutely require an opening through the coronal surface, and if you are incompetent to get this you must retrace your steps and learn how, before attempting any treatment.

Having secured an opening in the line of the axis of the nerve canals, you may attempt the removal of the pulp. You will greatly facilitate this by placing in the cavity some antiseptic, like carbolic acid, for a day or two, because this will shrink and harden the tissue so that it can probably be extracted whole, instead of piecemeal. In the anterior teeth this getting out the pulp entire will not be difficult, but in bicuspids and molars you will find that, with all your efforts, you cannot gain an entrance into some of the roots, because of the minuteness of the opening. When that is the case, it is well to give plenty of time to the treatment, after all that it is possible to get out has been removed. Keep the cavity aseptic by means of a pledget of cotton dipped in some antiseptic solution, and carefully sealed up with wax, or gutta-percha, or sandarach, changing it as often as is necessary, and in a few days that tissue which was left will have been sloughed out, and the pulp chamber and canals will be ready for filling.

Remember that all this is applicable only in those cases in which putrefactive changes have not been set up; those which have never been in a particularly septic condition. Of course, in your removal of the pulp you will use such instruments and appliances as best commend themselves to your judgment. Some employ barbed nerve broaches, some a hooked broach, some entangle the pulp in cotton wound on a broach. Use that which you find the most effective in your hands, but keep one thing always in mind: In thrusting a broach of any kind into a nerve canal, there is danger that you may go through an open foramen and wound the delicate tissue beyond the tooth, and yet greater liability to push some of the dead pulp or debris through, and thus have a foreign substance breeding disturbance which you cannot get at.

As to the antiseptic to be used in the treatment of nerve canals, you must use your own judgment founded on a knowledge of the peculiar qualities of each. A solution of Iodoform in Chloric Ether is excellent, (Pulverized Iodoform gr. v, Chloric Ether ℥j). Some prefer carbolic acid and glycerine, equal parts, and this gives very satisfactory results. Others employ Eucalyptus. Try these preparations for yourself, and then you will be able to judge of their merits. But whatever the preparation employed, always remember that it is likely to fail if it be mixed with saliva. Always put on the rubber dam before opening the cavity. Go to work like a professional man. Don't be a slovenly bungler and daub your remedy all over the patient's mouth. You need not expect good results unless you proceed in a workmanlike manner. The rubber dam is essential to a perfect success.

Of the filling of nerve canals I do not propose now to speak. My space is exhausted, and I have not said to you half what I should be glad to say. But I cannot hope to teach you all that you should know, even were I competent. I can only enlighten you a little concerning principles. Their application you must learn by thoughtful experiment. If, however, you will turn to the number of the INDEPENDENT PRACTITIONER for May, 1884, you will find my views concerning the filling of nerve canals very fully set forth in an editorial article.

Alveolar Abscess is also quite another matter, although many of the principles here laid down are applicable in its consideration. It may possibly form the subject for another letter, provided your patience be not already quite exhausted.

